# External Human–Machine Interfaces Can Be Misleading: An Examination of Trust Development and Misuse in a CAVE-Based Pedestrian Simulation Environment

**DOI:** 10.1177/0018720820970751

**Published:** 2020-11-26

**Authors:** Anees Ahamed Kaleefathullah, Natasha Merat, Yee Mun Lee, Yke Bauke Eisma, Ruth Madigan, Jorge Garcia, Joost de Winter

**Affiliations:** 1 2860 Delft University of Technology, The Netherlands; 2 414566 4468 University of Leeds, United Kingdom

**Keywords:** automated driving, pedestrians, external human–machine interfaces, trust, misuse, risk perception

## Abstract

**Objective:**

To investigate pedestrians’ misuse of an automated vehicle (AV) equipped with an external human–machine interface (eHMI). Misuse occurs when a pedestrian enters the road because of uncritically following the eHMI’s message.

**Background:**

Human factors research indicates that automation misuse is a concern. However, there is no consensus regarding misuse of eHMIs.

**Methods:**

Sixty participants each experienced 50 crossing trials in a Cave Automatic Virtual Environment (CAVE) simulator. The three independent variables were as follows: (1) behavior of the approaching AV (within-subject: yielding at 33 or 43 m distance, no yielding), (2) eHMI presence (within-subject: eHMI on upon yielding, off), and (3) eHMI onset timing (between-subjects: eHMI turned on 1 s before or 1 s after the vehicle started to decelerate). Two failure trials were included where the eHMI turned on, yet the AV did not yield. Dependent measures were the moment of entering the road and perceived risk, comprehension, and trust.

**Results:**

Trust was higher with eHMI than without, and the −1 Group crossed earlier than the +1 Group. In the failure trials, perceived risk increased to high levels, whereas trust and comprehension decreased. Thirty-five percent of the participants in the −1 and +1 Groups walked onto the road when the eHMI failed for the first time, but there were no significant differences between the two groups.

**Conclusion:**

eHMIs that provide anticipatory information stimulate early crossing. eHMIs may cause people to over-rely on the eHMI and under-rely on the vehicle-intrinsic cues.

**Application:**

eHMI have adverse consequences, and education of eHMI capability is required.

## Introduction

Pedestrian deaths constitute 16% of all traffic fatalities, and the vast majority of these crashes are due to human error (National Highway Traffic Safety Administration [NHTSA], 2019). Automated vehicles (AVs) have the potential to improve road safety by excluding the human driver from the control loop (Fagnant & Kockelman, 2015). In current traffic, when pedestrians decide to cross the road in front of an approaching vehicle, they rely on both implicit and explicit cues. Implicit cues are regular vehicle behaviors, such as speed and deceleration. Explicit cues, on the other hand, are not part of vehicle behavior, and include amongst others, eye contact, posture, and hand gestures of the driver (Sucha et al., 2017). One of the challenges in future AVs will be that the driver may be inattentive or even absent, which implies that explicit communication will become cumbersome or impossible.

External human–machine interfaces (eHMIs) may be a viable substitute for explicit communication in current traffic. More specifically, an eHMI could indicate the yielding intention of an AV through lights, symbols, text messages, or even sound (Ackermann et al., 2019; Bazilinskyy et al., 2019; Bengler et al., 2020; Burns et al., 2019; Habibovic et al., 2018; Lagström & Lundgren, 2015; Schieben et al., 2019). Although eHMIs have been found to affect pedestrians’ road-crossing decisions (e.g., Kooijman et al., 2019), other studies suggest that pedestrians rely predominantly on implicit cues, such as the AV’s approach speed and closing distance. eHMIs may, therefore, only provide a secondary source of information for pedestrians, which could be employed in ambiguous situations, for example (Clamann et al., 2017; Dey & Terken, 2017; Moore et al., 2019; Rodríguez Palmeiro et al., 2018; Rothenbucher et al., 2016). The extent to which pedestrians benefit from explicit communication in the form of eHMIs versus implicit communication in the form of the vehicle movement itself (e.g., Moore et al., 2019) is not yet well understood and the subject of some debate.

An evaluation of various eHMIs by Löcken et al. (2019) has shown that eHMIs that were deemed clear, supportive, and easy to use (i.e., high pragmatic quality) yielded the highest levels of self-reported trust. A possible concern after prolonged exposure to reliable AVs and clear eHMIs is that pedestrians may start to trust and rely strongly on the eHMI, while ignoring implicit cues from the AV, that is, its actual yielding behavior. This, in turn, may cause misuse, defined by Parasuraman and Riley (1997) as relying uncritically on automation without recognizing its limitations or failing to monitor the automation’s behavior. In the context of eHMIs, we define misuse as a situation where the pedestrian walks onto the road without critically evaluating the eHMI’s instructions. Faas et al. (2020, p. 181) studied an eHMI that showed the status of automation and found that, after the experiment, some pedestrians expressed concerns about automation misuse: “informing about the vehicle’s automated driving mode might lead to overtrust in the vehicle’s capabilities among pedestrians, who might then be less attentive when encountering such a vehicle.” These concerns of misuse can be related to other human factors phenomena, such as errors of commission, defined as doing what an automated aid tells one to do, even when other available data suggest that the automation aid is not recommending a proper course of action (Skitka et al., 1999), or compliance, defined as the tendency to perform an action cued by an automation alert (Dixon et al., 2007; Meyer et al., 2014).

A seminal study on trust development in general was performed by Lee and Moray (1992). They investigated changes in operators’ task performance and self-reported trust when interacting with a semi-automatic pasteurization plant. Results indicated that the participants’ trust and performance increased as they became familiar with the system. However, after experiencing a fault, trust declined considerably. More recently, Holländer et al. (2019) investigated the effects of an eHMI conveying incorrect information; that is, the AV stopped, but a “halt” symbol was presented, or the AV maintained speed while a green walking symbol was presented. Results showed that the incorrect information caused a sharp decline in perceived trust and safety. Perceived trust and safety recovered directly afterwards, indicating that the incorrect information had no clear lasting effect in subsequent trials with a properly functioning eHMI.

Based on the above, it appears essential to assess the development of trust and possible misuse during repeated exposure to eHMIs. One factor that has not been studied so far concerns the interplay between explicit cues (i.e., the eHMI signal) and implicit cues (i.e., AV speed and distance). If pedestrians are relying predominantly on implicit cues, then eHMI misuse should be unlikely. Conversely, if participants rely strongly on the information provided by the eHMI while ignoring implicit cues, then there is a risk of misuse. More specifically, the risk is that the pedestrian crosses if the eHMI signals so, even when the AV does not yield for the pedestrian.

One critical variable in the examination of reliance on explicit communication versus implicit communication is the eHMI onset timing. de Clercq et al. (2019) showed that participants felt more inclined to cross when the eHMI (e.g., a text “WALK” or a front brake light) switched on before the AV started braking, compared with a condition without the eHMI. Although early onset timing stimulates pedestrians to cross early, it could also cause misuse if pedestrians do not line up the information provided by the eHMI with the AVs implicit cues (i.e., is the AV indeed slowing down?).

This study aimed to examine pedestrians’ trust development and potential automation misuse during repeated encounters with an AV equipped with an eHMI, with a specific focus on the effects of eHMI onset timing. It was hypothesized that participants would trust a vehicle with an eHMI more than a vehicle without an eHMI, but would lose that trust if the system failed (i.e., eHMI turned on, but the AV did not yield). Yet participants were expected to regain trust quickly (see Holländer et al., 2019). In real traffic, pedestrians could experience this type of “failure” if an AV stops for another pedestrian or object further down the road. We further expected that pedestrians would cross earlier when the eHMI provided information “early,” that is, when the eHMI onset occurred before the AV started braking. We compared this to an eHMI that provided “late” information, which means that the eHMI turned on after the AV started braking. Finally, we expected that pedestrians who have been repeatedly exposed to an early-onset eHMI would be more likely to initiate crossing when the eHMI failed, as compared with pedestrians who have repeatedly encountered a late-onset eHMI.

Previous research found that for an AV that aims to communicate “I am giving way,” a pulsing light band and conventional flashing headlights were more preferred than a pulsing lamp or a light band that filled from front to back (Lee et al., 2019). It has further been recommended that, for better visibility, eHMIs need to be positioned on the front and the sides of the car instead of only on the front (Eisma et al., 2020). Our study featured an AV with a 360° pulsing light band located on the front and sides of the vehicle as well as on the grill. This positioning should enable the pedestrian to be aware of the light band from any direction. The experiment was conducted in a Cave Automatic Virtual Environment (CAVE) simulator at the Institute for Transport Studies, University of Leeds. A realistic simulation environment was regarded as important for accurate risk perception and the assessment of potential misuse.

## Methods

### Participants

Sixty participants (30 males and 30 females) aged between 18 and 35 years (*M* = 24.4, *SD* = 4.0) took part in the study. All participants were recruited via posters at the University of Leeds student union, through acquaintances, or via posts on social media. The 60 participants comprised of 15 different nationalities, mostly British (28), Chinese (6), Malaysian (5), Lithuanian (3), French (3), Polish (3), and Spanish (3). The participants were either students or university employees.

Twenty-nine participants were used to left-hand traffic, 15 participants were used to right-hand traffic, and the remaining 16 participants were used to both left- and right-hand traffic. Twenty-one participants wore their glasses during the experiment. Thirty-seven participants had experience with head-mounted virtual reality.

This research complied with the American Psychological Association Code of Ethics and was approved by the University of Leeds Research Ethics Committee (Ref: LTTRAN-097). All participants provided written informed consent.

### Pedestrian Simulator

**Figure 1:**
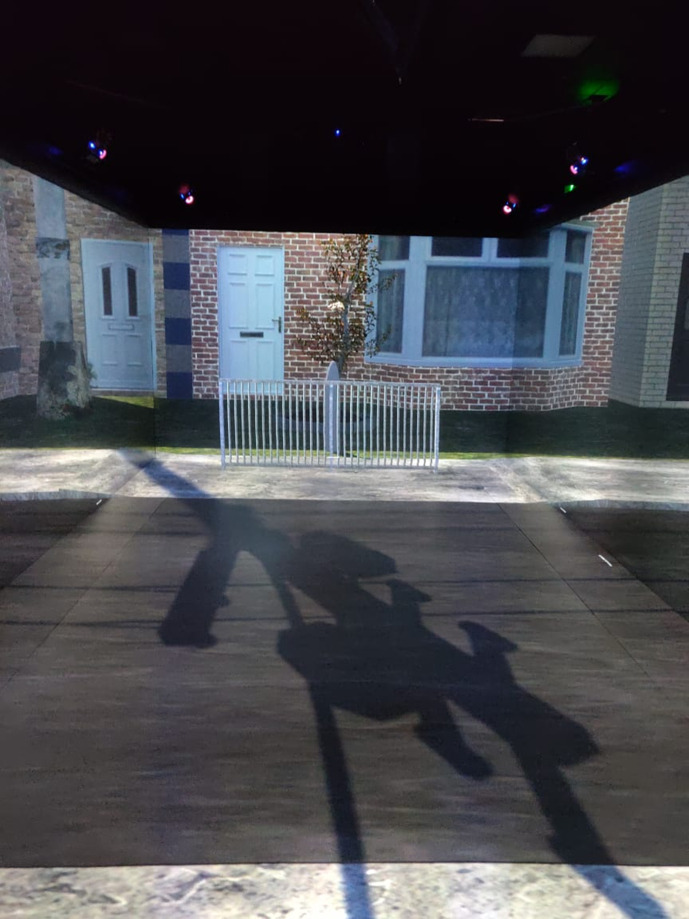
Participant’s view of the pedestrian simulator.

The study was conducted in the Highly Immersive Kinematic Experimental Research (HIKER) pedestrian simulator (Figure 1) at the University of Leeds, which is a 9-m long by 4-m wide CAVE-type simulator. Participants wore stereoscopic motion-tracking glasses and could move around freely. The virtual environment featured a single-lane, 4.19-m-wide road in a city environment during daytime (Figure 1). A fence was placed on the other side of the road, to prevent the participant from crossing beyond that point. The simulator was programmed to alert the participant with a warning sound when they were too close to the wall beyond the fence.

The experiment ran on a computer rack of eight machines, each one of them containing an Intel^®^ Core™ i9-7900X CPU @ 3.30 GHz processor, 128 GB RAM, and 8 GB Nvidia Quadro P4000. The scenario was generated in Unity 2017.4.17 with a Middle VR 1.7.1.2 licensed plugin and ran in stereo mode at a resolution of 2560 × 1600 pixels per projector. Ten IR motion trackers connected to the software Vicon Tracker 3.7 tracked the position and head angle of the participant.

### Experimental Design

The experiment had three independent variables: eHMI onset, yielding behavior of the AV, and eHMI presence.

#### eHMI onset

eHMI onset was a between-subjects variable with two levels: −1 s and +1 s. Participants were alternately assigned to the Group + 1 and Group −1. For the +1 Group, the eHMI turned on 1 s after the vehicle started to decelerate. For the −1 Group, the eHMI turned on 1 s before the vehicle started to decelerate. The eHMI onset timing offsets were based on de Clercq et al. (2019), who found that a −1 s timing yielded substantial benefits to pedestrians, in the sense that pedestrians were willing to cross the road earlier with the eHMI compared with without the eHMI. The +1 timing offset also had benefits, although smaller as compared with the −1 s timing.

#### Yielding behavior

**Figure 2:**
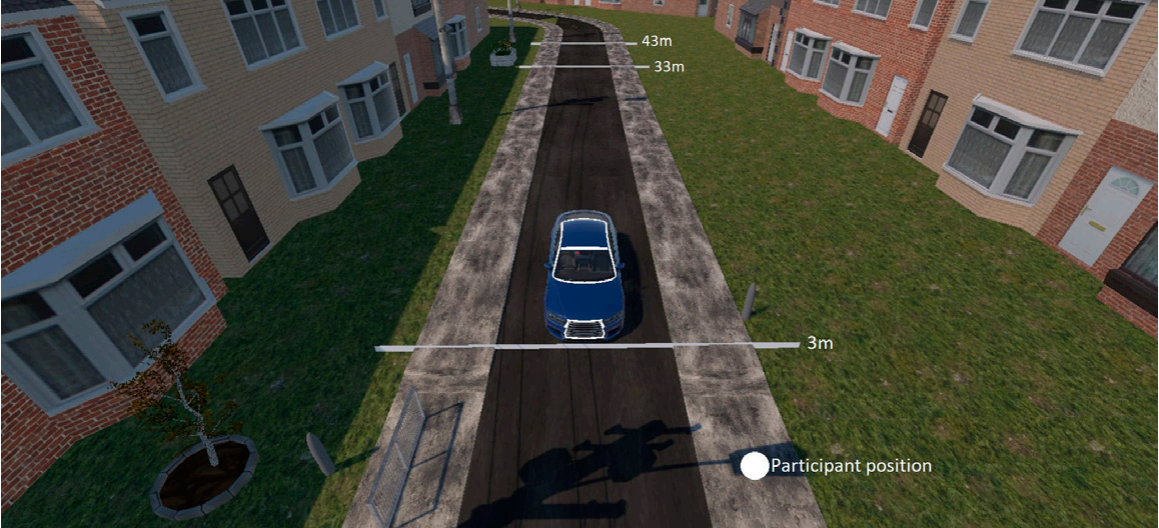
The lines display the distances where the vehicle started to decelerate (33 m and 43 m) and the complete stop at 3 m from the participant. The white circle indicates the participant’s initial position. The white lines and circles were not visible during the experiment.

**Figure 3:**
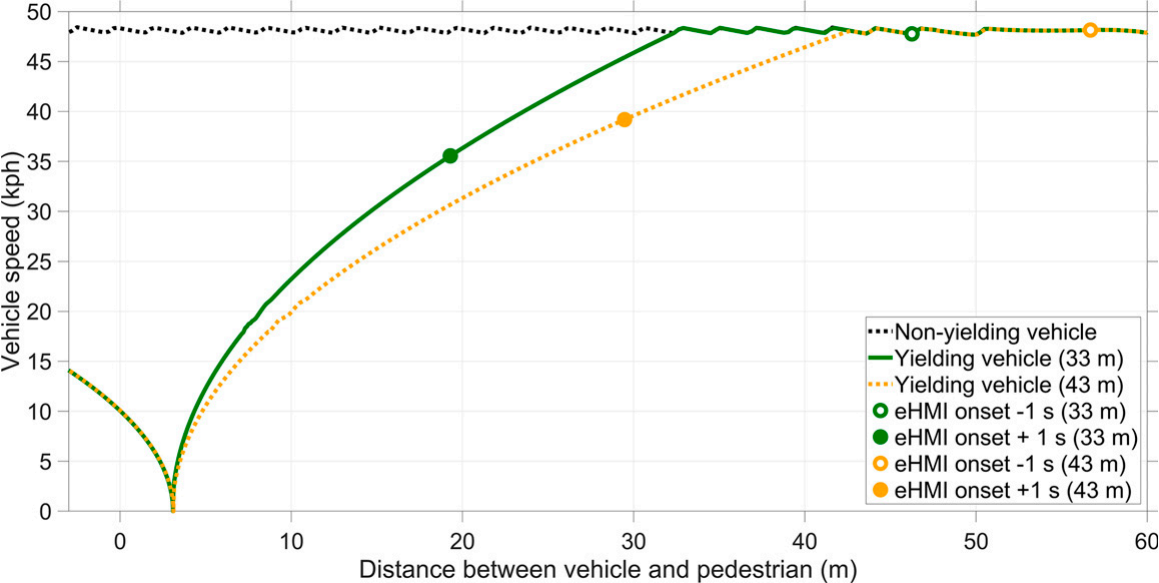
Vehicle speed as a function of the distance between the automated vehicle and the pedestrian. The distance between the pedestrian and the approaching vehicle is defined along the x axis (i.e., parallel to the direction of the road). Note that the automated vehicle drives past the pedestrian when the distance is 0 m. eHMI = external human–machine interface.

Yielding behavior was a within-subject variable with three levels: (1) yielding while starting to decelerate at a 33 m distance, (2) yielding while starting to decelerate at a 43 m, and (3) no yielding, as illustrated in Figure 2. The interactions with the eHMI occurred during yielding trials only. The nonyielding trials were included to make participants aware that they could not cross the road in all trials but would be required to look at the AV before crossing. During nonyielding trials, the AV maintained a speed of 30 mph (~48 kph) without stopping. For the yielding trials, the AV decelerated at 2.24 m/s^2^ and 2.99 m/s^2^ for stopping distances of 43 m and 33 m, respectively. We used two stopping distances instead of one, to introduce variability and prevent participants from recognizing that the AV always behaved in the same way. During yielding trials, the vehicle came to a stop at a distance of 3 m from the participant and waited until the pedestrian had crossed; the car then drove off again. If the participants crossed before the vehicle came to a complete stop, the car accelerated again without stopping. Figure 3 shows the speed versus distance relationships for the two stopping distances together with markers that indicate the eHMI onsets.

#### eHMI presence

The eHMI presence was a within-subject variable, with two levels: eHMI off and eHMI on. In all trials, the vehicle arrived around the corner in an identical manner with the eHMI turned off. In yielding trials, the eHMI turned on in 75% of the cases (i.e., eHMI on trials) and remained off in 25% of the cases (i.e., eHMI off trials). In nonyielding trials, the eHMI remained off. Accordingly, the experiment mimicked a mixed-traffic situation with some vehicles having no eHMI and some vehicles having an eHMI to indicate when the vehicle is yielding.

**Figure 4:**
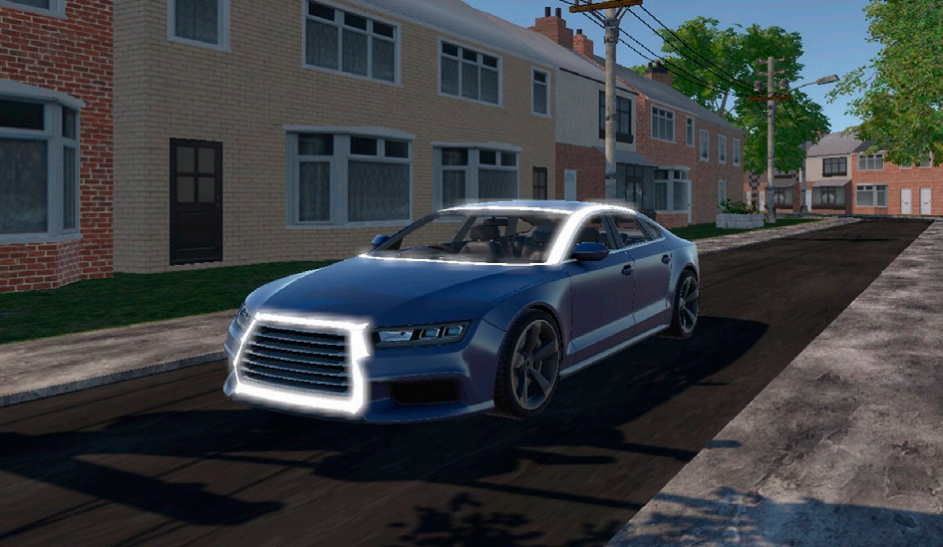
Automated vehicle with the eHMI at 100% intensity. The vehicle had a width of 1.90 m and a length of 4.96 m. eHMI = external human–machine interface.

In pilot tests, we noticed that our initial light band design, as proposed by Lee et al. (2019), was sometimes not noticed by participants. Because our aim was to evaluate possible misuse of the eHMI, we increased the brightness of the eHMI so that participants would not be likely to overlook it. Accordingly, we designed an eHMI consisting of a thick white light band around the top edges of the car and the front grill (Figure 4). The light band was pulsating to attract attention. More specifically, when the light band was on, its intensity varied in a zigzag-like manner between 30% and 100%, with an intensity peak-to-peak interval of 0.80 s. That is, the intensity was 100% at the onset of the eHMI, intensity decreased linearly to 30% in 0.28 s, stayed at a constant level of 30% for 0.24 s, and increased linearly to 100% in 0.28 s again. The light band remained on until the moment the participant finished crossing.

Each participant completed 50 trials. In each trial, the participant encountered a blue AV approaching from the right. Once the participant had triggered the start of the trial, the vehicle appeared around the corner, initially out of sight, at a longitudinal and lateral distance from the pedestrian of 73 m and 22 m, respectively, and driving at a speed of 30 mph (48 kph).

Participants completed four blocks of trials, consisting of 12 trials each. There was one failure trial after Block 3, to examine initial misuse, and another one after Block 4, to evaluate whether misuse persisted (Table 1). In the failure trial, the eHMI turned on, but the AV continued to drive at a constant speed. Table 2 shows the number of trials per yielding condition within each block. The order of trials was random within each block, for all blocks, and different for each participant.

**Table 1:** Experimental Blocks

Block 1	Block 2	Block 3	Failure Trial 1	Block 4	Failure Trial 2
Trials 1–12	Trials 13–24	Trials 25–36	Trial 37	Trial 38–49	Trial 50

**Table 2:** Number of Trials Per Yielding Condition and eHMI Presence Condition, Per 12-Trial Block

Distance Between Pedestrian and Vehicle at the Onset of Deceleration	eHMI Presence	Number of Trials per Block
33 m	On	3
43 m	On	3
33 m	Off	1
43 m	Off	1
No yielding	Off	4

*Note*. eHMI = external human–machine interface.

In the two failure trials, the eHMI switched on at 38 m (average onset distance of the two groups) from the participant, but the vehicle did not yield. The eHMI timing for the failure trial was the same for Groups +1 and -1.

### Dependent Measures

**Figure 5:**
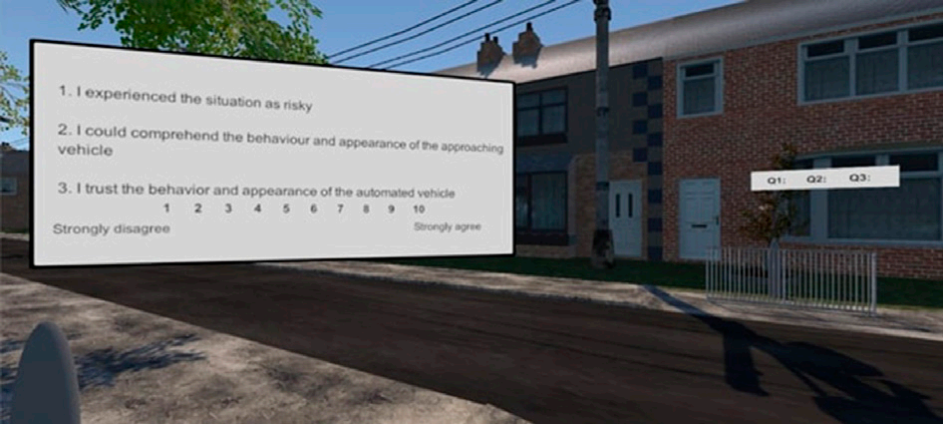
Post-trial questions displayed on the participant’s left. Each question was answered verbally on a 10-point Likert scale from 1 (*Strongly Disagree*) to 10 (*Strongly Agree*).

The dependent measures included measures assessing perceived risk, comprehension, and trust. More specifically, the following three questions were displayed to participants on the HIKER lab screen, after each trial (Figure 5):

1. I experienced the situation as risky.
2. I could comprehend the behavior and appearance of the approaching vehicle.
3. I trust the behavior and appearance of the automated vehicle.

Response to these was verbal and recorded by the experimenter.

Additionally, we recorded the pedestrian’s position along an imaginary axis that runs perpendicular to the road as a function of elapsed time since the start of the trial. Positions of 0 m, 2.05 m, and 6.24 m correspond to the starting point, the nearest edge of the road, and the farthest edge of the road, respectively.

The following dependent measures were computed:

- Moment of entering the road in seconds since the start of the trial (s). This measure was computed only for trials in which the AV was yielding for the participant, because in nonyielding trials, the participants almost never entered the road.
- Pedestrian position when the car passed (*m*). For each trial in which the AV maintained speed, we recorded the pedestrian’s position at the moment the car passed the pedestrian. From this measure, we computed the percentage of participants who were on the road at the moment the car passed.

### Procedure

After the participants entered the HIKER room, the researcher explained that the experiment was aimed at studying the crossing behavior of pedestrians when interacting with AVs. Next, the participants read and signed the informed consent form. The form mentioned that if the eHMI is on, it means that the car is yielding, and if the eHMI is off, the car sometimes yields and sometimes continues driving without yielding. The task instructions were described as follows: “With the glasses on, you will look to the corner on the right and take one or two steps forward. The car will then appear from the right corner. You will then move forward along the pavement and decide to cross, or not, depending on the yielding behavior of the vehicle.”

Next, participants were asked to complete a demographic questionnaire and a practice session. They were verbally briefed by the researcher in the simulator on how to trigger the onset of the car, to cross the road, and answer the post-trial questions The practice trials consisted of five trials: three trials with a nonyielding vehicle, one trial with the eHMI on with a yielding vehicle (−1 s, 33 m), and one trial with the eHMI off, with a yielding vehicle (33 m).

Participants initially stood on the edge of the pavement in the simulator. The approach of the AV was automatically triggered based on the participant’s head angle and position in the simulator. The participant then moved forward and decided whether or not to cross in front of the approaching AV. After crossing, the participants returned to the starting position and verbally answered the three post-trial questions that appeared on their left, as shown in Figure 5. Once they provided a response to the questions, they were ready to start with the next trial.

After the experiment, the participants completed a post-experiment questionnaire. The post-experiment questionnaire consisted of questions regarding the experiment in general and a set of virtual presence questions (Witmer & Singer, 1998). They were then reimbursed with £10. The experiment lasted approximately 1 hr per participant.

### Participant Exclusion and Statistical Analyses

All data were post-processed in MATLAB R2019b. Tables were constructed containing the participants’ means and standard deviations of the scores of the dependent measures, separated per block, eHMI presence, and yielding behavior.

Comparisons for four dependent measures (perceived risk, comprehension, trust, and moment of entering the road) were performed for the −1 Group versus the +1 Group (averaged across all yielding trials of Blocks 1–3), using independent-samples *t*-tests. We used Fisher’s exact test for comparing the −1 Group with the +1 Group, regarding the number of participants who were on the road when the car passed during Failure trials 1 and 2. Additionally, we compared the eHMI on and eHMI off conditions (averaged across all yielding trials of Blocks 1–3), using paired *t*-tests. The scores for Block 3 versus Block 4 were also compared, using paired *t*-tests. For all statistical tests, we used an alpha level of .05. Selecting an alpha value always involves a trade-off between preventing false positives and false negatives (e.g., Mudge et al., 2012). The reason for choosing .05 instead of a more conservative number is that we wanted to prevent false negatives regarding the effects of eHMIs. We used *t*-tests, as opposed to more complex (multivariate) tests, because the use of a *t*-test is consistent with our hypotheses, which address main effects and not interactions.

## Results

In each trial, the AV approached from a blind curve. This road layout ensured that participants could not see the AV arrive from a distance and was intended to prevent them from crossing the road in front of a nonyielding AV or before a yielding AV started to decelerate. An inspection of the results showed that some participants were on the road or had already crossed the road at the moment the nonyielding AV passed. According to the experimenter’s notes and inspection of the raw data, these participants walked quickly, sometimes without slowing down. They often crossed directly in front of the nonyielding AV, and in some cases were run over by it. These behaviors were regarded as unrealistic (in real traffic, pedestrians do not cross that hazardously or walk under cars) and against the intention of the experiment (the experiment was designed so that participants would not cross in front of nonyielding AVs). For these reasons, it was decided to screen out participants. More specifically, participants who were on the road or had already crossed the road at the moment the nonyielding AV passed in more than 6 out of 18 nonyielding trials (i.e., four trials per block plus two failure trials) were excluded from all analyses. Accordingly, five participants from the +1 Group, and two participants from the −1 Group were excluded. They had walked onto the road 17, 10, 10, 14, and 15 out of 18 times (+1 Group), and 7 and 10 out of 18 times (−1 Group). The +1 Group consisted of 25 participants (12 males and 13 females), aged between 19 and 34 years (*M* = 24.9, *SD* = 3.8). The −1 Group consisted of 28 participants (13 males and 15 females), aged between 19 and 35 years (*M* = 24.0, *SD* = 4.2). For completeness, the Supplemental materials contain the results of all statistical tests for the full sample (30 participants in the +1 Group and 30 participants in the −1 Group). The results are generally the same, except for the higher standard deviations of the road entering times for the full sample as compared with the reduced sample (see Supplemental materials).

### Comparisons Between eHMI on (−1 Group & +1 Group) and eHMI Off

**Figure 6:**
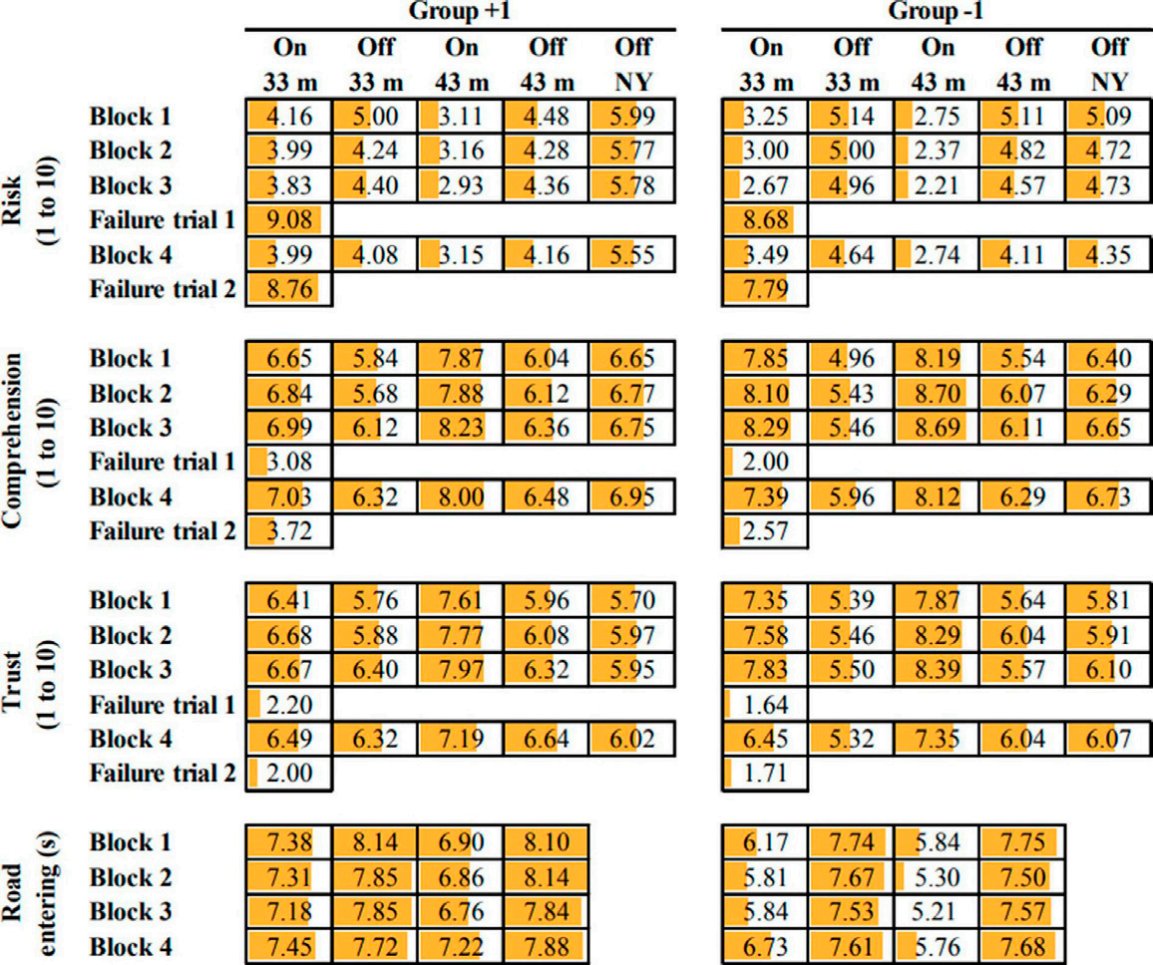
Means of the participants’ results per dependent measure and experimental condition. The cells for Risk, Comprehension, and Trust are filled linearly according to the depicted mean value on a scale from 1 (no orange in the cell) to 10 (entire cell orange). The cells for the road entering time are filled linearly according to the depicted mean value on a scale from 5 s to 8 s. Standard deviations, reflecting the magnitude of individual differences, are available in the Supplemental materials. NY = no yielding.

Figure 6 shows the mean scores of the dependent measures, separated per block (1–4), eHMI presence (on, off), and yielding behavior (33 m, 44 m, or no yielding). For yielding AVs, perceived risk was lower, and perceived comprehension and trust higher, when the eHMI was on compared with off. The differences in risk, comprehension, and trust between the eHMI on and off conditions (averaged across all yielding trials of Blocks 1–3) were significant for the −1 Group (*t*(27) = −6.44, *p* < .001, *t*(27) = 7.11, *p* < .001, *t*(27) = 5.80, *p* < .001, respectively) and for the +1 Group (*t*(24) = −2.81, *p* = .010, *t*(24) = 4.83, *p* < .001, *t*(24) = 3.68, *p* = .001, respectively). Furthermore, Figure 6 shows that the perceived risk was generally lower, and the comprehension and trust higher, in the −1 Group as compared with the +1 Group. The results of independent-samples *t*-tests for the three respective measures (risk, comprehension, and trust) between the +1 Group and −1 Group were as follows: *t*(51) = 1.80, *p* = .078, *t*(51) = −2.47, *p* = .017, *t*(51) = −1.69, *p* = .098 (averaged across all eHMI on trials of Blocks 1–3).

**Figure 7:**
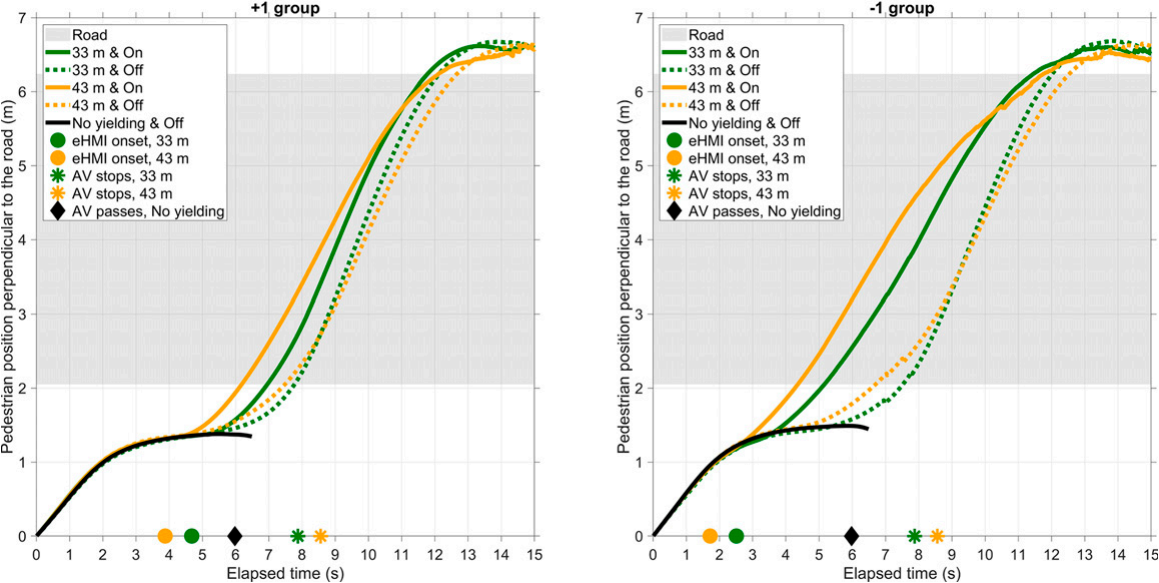
Mean pedestrian position perpendicular to the road as a function of elapsed time per group, yielding condition, and eHMI condition (Blocks 1–3 only). Also shown are the moments the eHMI turned on, the moments the vehicle came to a stop, and the moment the vehicle passed in the eHMI off trials. AV = automated vehicle; eHMI = external human–machine interface.

Figure 6 further shows that pedestrians entered the road earlier for the eHMI on as compared with the eHMI off condition. The differences between eHMI on and off conditions (averaged across all yielding trials in Blocks 1–3) were significant for both the −1 Group (*t*(27) = −7.26, *p* < .001) and the +1 Group (*t*(24) = −7.46, *p* < .001). Furthermore, according to an independent-samples *t*-test, the road entering time was earlier for the −1 Group than for the +1 Group, *t*(51) = 3.07, *p* = .003 (averaged across all eHMI on Trials of Blocks 1–3). These effects are illustrated in Figure 7, showing the mean pedestrian position as a function of elapsed time for all yielding conditions.

### Dynamics of Risk, Comprehension, Trust, and Time of Entering the Road (eHMI on Trials)

**Figure 8:**
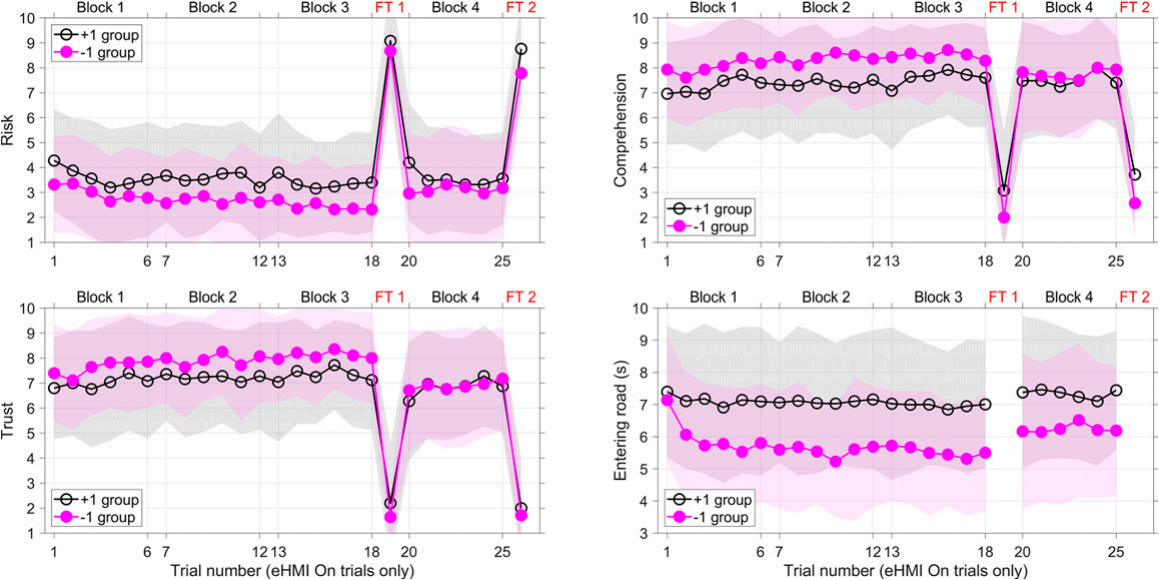
Mean values of perceived risk of the situation (left top), comprehension of the behavior and appearance of the vehicle (right top), trust in the behavior and appearance of the vehicle (left bottom), and the moment the pedestrian entered the road (right bottom). The horizontal axis is the trial number, only counting the trials where the eHMI was on. At trial numbers 19 and 26, the eHMI failure occurred. The shaded areas represent the mean ± standard deviation, depicted in gray for the +1 Group and in light magenta for the −1 Group (overlap shows up as a darker magenta shading). eHMI = external human–machine interface; FT 1 = Failure Trial 1; FT 2 = Failure Trial 2.

Figure 8 illustrates that participants learned the functioning of the eHMI during the first three blocks, as indicated by a slight decrease of perceived risk and a decreased time for entering the road.

Figures 6 and 8 show that, for both groups, in Failure Trial 1 (post Block 3) and Failure Trial 2 (post Block 4), perceived risk showed a substantial increase, and comprehension and trust a considerable decrease, as compared with the previous trials. The participants’ mean risk levels for failure trials were as high as 7.79–8.88 on a scale of 1 to 10. Trust, on the other hand, was low (between 1.64 and 2.38). Independent-samples *t*-tests of the perceived risk, comprehension, and trust for the failure trials showed no significant differences between the +1 Group and the −1 Group for Failure Trial 1 (*t*(51) = .75, *p* = .456, *t*(51) = 1.50, *p* = .139, *t*(51) = 1.13, *p* = .265, respectively) and Failure Trial 2 (*t*(51) = 1.47, *p* = .147, *t*(51) = 1.68, *p* = .099, *t*(51) = .63, *p* = .530, respectively).

Figures 6 and 8 show that the scores on the dependent measures recovered immediately after the first failure trial. We examined the differences between block 3 (before Failure Trial 1) and Block 4 (after Failure Trial 1). Figure 6 shows that in block 4, perceived risk was generally higher, and comprehension and trust lower, as compared to Block 3 (averaged over the eHMI On trials). The participants in the −1 group entered the road later in block 4 as compared to block 3. according to a paired-samples *t*-test, the effects between Block 3 and 4 were as follows for the −1 group: *t*(27) = −3.15, *p* = .004, *t*(27) = 3.22, *p* = .003, *t*(27) = 3.85, *p* < .001, and *t*(27) = −3.84, *p* < .001, for risk, comprehension, trust, and time of entering the road, respectively). For the +1 Group, the Block 3 vs. Block 4 effects were: *t*(24) = −1.26, *p* = .220, *t*(24) = 0.52, *p* = .609, *t*(24) = 2.00, *p* = .057, and *t*(25) = −3.20, *p* = .004, respectively). To summarize, as can also be seen from Figure 8, participants of the −1 group in particular lost trust after experiencing the first failure trial.

### Walking Onto the Road in the Failure Trial

**Figure 9:**
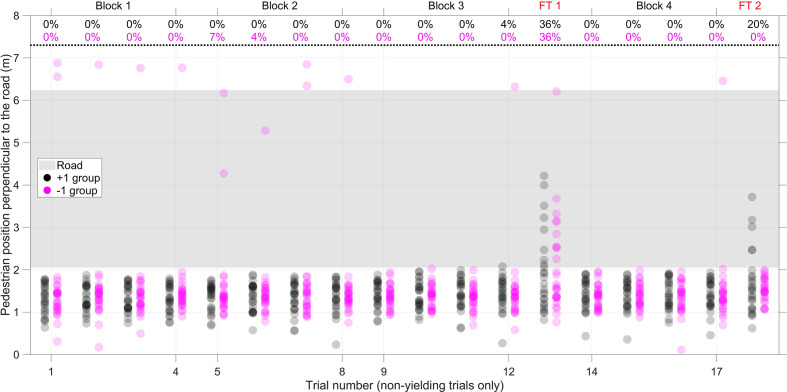
Pedestrian location at the moment the front of the car passed. The top of the figure shows the percentage of participants in the +1 Group (25 participants) and −1 Group (28 participants) who were on the road at the moment the car passed. The markers are transparent so that overlap can be distinguished. FT 1 = Failure Trial 1; FT 2 = Failure Trial 2.

From Figure 9, it can be seen that, before Failure Trial 1, three participants from the −1 Group (nonyielding trial no. 5 and 6) and one participant from the +1 Group (nonyielding trial no. 12) were on the road at the moment the AV passed. Thirty-six percent of the participants were on the road during the first failure trial. In the second failure trial, no participants from the −1 Group were on the road. In the +1 Group, 20% of the participants in Failure Trial 2 were standing/walking on the road while the vehicle passed.

We hypothesized that the +1 Group would yield lower misuse of the eHMI when compared with the −1 Group. For Failure Trial 1, nine of 25 participants in the +1 Group and 10 of 28 participants in the −1 Group who were on the road (*p* = 1.000 according to a Fisher’s exact test). For Failure Trial 2, five of 25 participants in the +1 Group and zero of 28 participants in the −1 Group were on the road, a significant effect that was contrary to our hypothesis (*p* = .019 according to a Fisher’s exact test).

**Figure 10:**
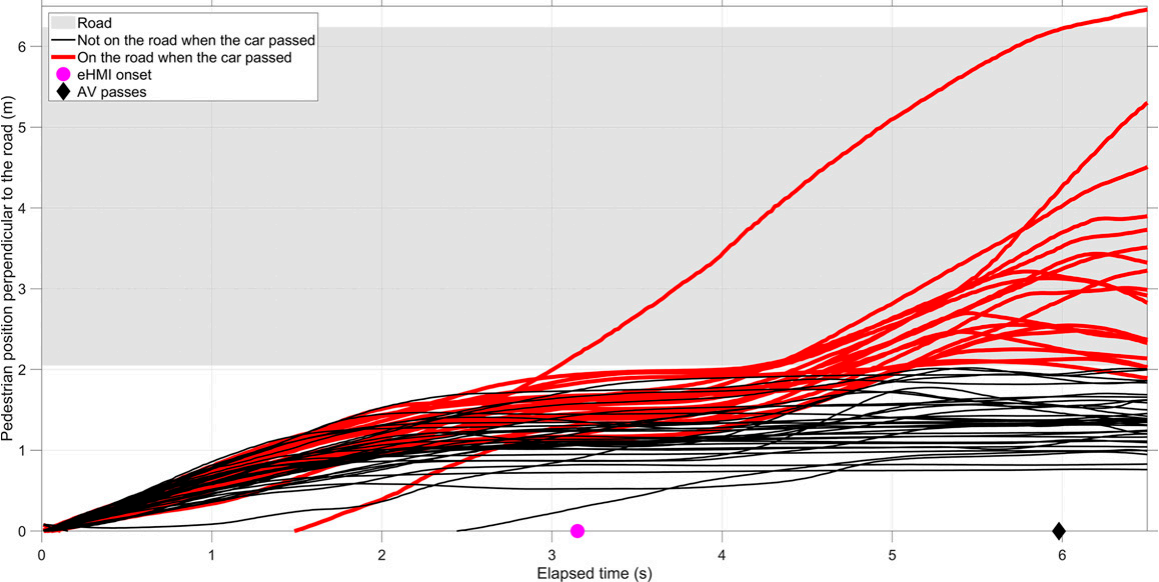
Mean pedestrian position perpendicular to the road as a function of elapsed time in Failure Trial 1. A distinction is made between pedestrians who were on the road when the car passed and pedestrians who were not on the road when the car passed. AV = automated vehicle; eHMI = external human–machine interface.

Figure 10 provides further insight into the behavior of participants in Failure Trial 1. Almost all participants walked up to the edge of the road and stopped, as can be seen from the horizontally running lines at an elapsed time of 3 s. After the eHMI turned on at 3.15 s, a portion of participants walked onto the road. Some participants noticed that the car was not stopping for them and stepped back, as indicated by the negatively slowing lines from an elapsed time of 5 s onward.

## Discussion

Millard-Ball (2018) argued that AVs will always be risk-averse, which may cause pedestrians to misuse the AVs by crossing the road in a careless manner. In the present study, we focused on misuse caused by AVs that are equipped with a salient eHMI in the form of a light band. We hypothesized that participants would trust the AV with an eHMI more than the AV without an eHMI, but would lose trust if the eHMI failed, yet trust would recover quickly. We further expected that pedestrians would cross earlier when the eHMI onset was early, as compared with late eHMI onset.

The hypotheses were all confirmed. The results showed that if the eHMI was on, participants in the −1 Group entered the road earlier, and reported higher trust, as compared with participants in the +1 Group. In turn, participants entered the road earlier and reported higher trust, higher comprehension, and lower risk, when the eHMI was on compared with when it was off. These results replicate earlier research (de Clercq et al., 2019; Faas et al., 2020) that showed that after a short instruction/training, eHMIs induce trust and stimulate pedestrians to cross, especially if the eHMI turns on before the vehicle starts to decelerate. Earlier studies suggest that a light-based eHMI is initially incomprehensible to users, but that people can learn its meaning with only a few trials of practice (de Clercq et al., 2019; Habibovic et al., 2018; Hensch et al., 2020). The high comprehension ratings from the very start of the experiment (Figure 8, right top) indicate that the light band eHMI was indeed easily understood.

The first failure trials had strong effects, in the sense that in as much as 35% of the trials, participants inadvertently entered the road, and the majority of participants expressed feeling a high level of risk. The results for perceived trust are consistent with Holländer et al. (2019) and Lee and Moray (1992), who found that when participants experienced a failure of the automated system, their trust declined strongly. As shown in Figure 9, some participants walked more than 1 m onto the road when the AV drove by, which meant the AV crashed into them. Figure 10 illustrates that these participants started walking immediately after the eHMI turned on and realized too late that the AV would not slow down. In other words, roughly one-third of the participants relied on the explicit communication of the eHMI while initially ignoring the vehicle-intrinsic information, whereas the remaining participants appeared to rely more strongly on the AV’s implicit communication for deciding whether to step onto the road. The perceived risk, comprehension, trust, and time of entering the road recovered immediately after the failure trial. However, compared with the crossing trials before the failure trial, significant differences remained, especially for the −1 Group. In other words, participants of the −1 Group had lower trust after the first failure trial, as compared with before it.

We hypothesized that pedestrians who had previously encountered an eHMI that provided “early” information (−1 Group) would be more likely to enter the road during failure trials as compared with those who were exposed to the late eHMI onset timing (+1 Group). However, our results showed that there were no significant differences between the two groups during Failure Trial 1. During Failure Trial 2, participants of the +1 Group were more likely to enter the road as compared with participants from the −1 Group, which was contrary to our hypothesis. While this effect needs to be replicated, we speculate that the reason why nobody in the −1 Group crashed in Failure Trial 2 is that participants of the −1 Group had learned from Failure Trial 1 that they should wait a little to verify from implicit cues that the AV is indeed slowing down to a stop. This speculation is supported by Figure 8, showing that participants in the −1 Group entered the road later after Failure Trial 1 as compared with before. For the +1 Group, on the other hand, the eHMI during the non-failure-trials turned on relatively late, and participants may have had too little time to (learn to) wait in order to verify the validity of the eHMI signal and benefit from the eHMI at the same time. To summarize, it is possible that, after having experienced a failure trial, the −1 Group started to rely more prominently on the implicit communication of the AV, while some participants of the +1 Group continued to rely on the explicit eHMI signal.

## Strengths and Limitations

This study was conducted in a CAVE simulator, where participants were able to move freely and could also see their own body in the environment, something that is not possible in most simulators that use head-mounted displays. This is a likely explanation for the high presence ratings regarding “proficiency in moving and interacting with the virtual environment” (see the Supplemental Materials). A limitation of our study is that the simulator did not include sound. Furthermore, some participants verbally commented that the display resolution and quality were not great. This limitation can be explained by the stereo glasses themselves, and the fact that the stereo simulation could be only run at a slightly reduced resolution than the achievable 4K resolution by all eight projectors. Another limitation is that our study involved only one AV in a single lane, and therefore did not examine eHMI misuse in cases where pedestrians have to distribute their visual attention among multiple actors.

## Conclusions and Recommendations

The results from this study clearly show that pedestrians are prone to misusing an eHMI after repeated exposure to that eHMI. In other words, there is a risk that pedestrians will “blindly” follow-up the eHMI’s message and ignore the implicit communication of the AV. However, contrary to our expectations, the eHMI onset timing did not appear to cause much difference when it comes to the degree of misuse during the first failure trial. This study also showed that when an eHMI signals the intent of the AV before the AV starts to decelerate, the eHMI causes the pedestrians to cross earlier, as compared with an eHMI that turns on after the vehicle starts to decelerate.

For future research, a more fine-grained understanding is required about whether pedestrians base their crossing decisions on implicit cues from the AV or explicit cues from the eHMI. Such a study could be conducted by using, for example, eye-gaze-contingent methods or psychophysics techniques. Furthermore, we recommend that pedestrians in future traffic should be made aware of the capabilities of the automated driving system, especially if the automation is prone to malfunction. For example, pedestrians could be made aware that an eHMI might turn on when the AV detects a pedestrian but will not yield for that pedestrian (but for another pedestrian instead). The present study showed that this type of failure caused some pedestrians to crash with the AV. It may be expected that the autonomous emergency braking (AEB) systems of future AVs will be able to alleviate the impact, although the extent to which this is possible would depend on how early the AV can detect and predict the pedestrian’s crossing intentions (Kooij et al., 2019). Education could help pedestrians maintain a calibrated amount of trust and could prevent misuse and disuse of the automated driving system. For example, pedestrians may be taught that eHMIs do not necessarily address the pedestrian himself, but could also address other pedestrians or road elements, such as a zebra crossing further down the road (and see Boelhouwer et al., 2020, for relevant recommendations regarding information-provision for in-vehicle automation systems). Finally, whether the present findings generalize to real traffic environments with a more diverse pool of participants remains unknown. One concern is that in real traffic, where pedestrians are not instructed or trained, they might simply overlook the led strips on the AV (Cefkin et al., 2019). It is recommended that future studies examine long-term trust development as well as traffic with multiple cars and multiple pedestrians of different age groups.

## Key Points

- This study examined pedestrians’ trust development and misuse during repeated encounters with an AV equipped with an eHMI that signaled the AV’s yielding intention.
- Pedestrian crossed the road earlier and exhibited higher perceived trust when the eHMI was on, compared with when it was off.
- An eHMI variant that turned on before the vehicle started to decelerate stimulated early crossing.
- Misuse was evaluated in failure trials, in which the eHMI turned on but the AV did not yield.
- Misuse occurred among 35% of participants in the first failure trial.

## Supplemental Material

Supplementary data - Supplemental material for External Human–Machine Interfaces Can Be Misleading: An Examination of Trust Development and Misuse in a CAVE-Based Pedestrian Simulation Environment Click here for additional data file. Supplemental material, Supplementary data, for External Human–Machine Interfaces Can Be Misleading: An Examination of Trust Development and Misuse in a CAVE-Based Pedestrian Simulation Environment by Anees Ahamed Kaleefathullah, Natasha Merat, Yee Mun Lee, Yke Bauke Eisma, Ruth Madigan, Jorge Garcia and Joost de Winter in Human Factors: The Journal of Human Factors and Ergonomics Society
