# Supplementary material for: External Human–Machine Interfaces Can Be Misleading: An Examination of Trust Development and Misuse in a CAVE-Based Pedestrian Simulation Environment
Source: Hum Factors. 2020 Nov 26;64(6):1070–85. doi: 10.1177/0018720820970751 (PMC9421345; doi:10.1177/0018720820970751)
Supplement: Supplementary data - Supplemental material for External Human–Machine Interfaces Can Be Misleading: An Examination of Trust Development and Misuse in a CAVE-Based Pedestrian Simulation Environment [file sj-pdf-1-hfs-10.1177_0018720820970751.pdf]

Supplementary Materials

The tables below show the results of the statistical tests as reported in the paper ($N = 53$) and for the full sample of participants ($N = 60$). It can be seen that statistical significance ($p < 0.05$) is the same, with two exceptions highlighted in yellow. The lack of significance for the road entering time between the +1 Group vs the -1 Group can be attributed to the increased standard deviations for the full sample.

Table S1

Overview of statistical tests reported in the paper (N = 53) and for the full sample (N = 60). Differences in statistical significance (p < .05) are highlighted in yellow.

		On vs. Off			
		Group+1		Group-1	
		**With exclusions (N = 53)**	**Without exclusions (N = 60)**	**With exclusions (N = 53)**	**Without exclusions (N = 60)**
**Risk (1 to 10)**	**Blocks 1-3**	*t* (24) = -2.81, *p* = .010	*t* (29) = -3.51, *p* = .001	*t* (27) = -6.44, *p* < .001	*t* (29) = -6.95, *p* < .001
**Comprehension (1 to 10)**	**Blocks 1-3**	*t* (24) = 4.83, *p* < .001	*t* (29) = 5.63, *p* < .001	*t* (27) = 7.11, *p* < .001	*t* (29) = 7.09, *p* < .001
**Trust (1 to 10)**	**Blocks 1-3**	*t* (24) = 3.68, *p* = .001	*t* (29) = 4.58, *p* < .001	*t* (27) = 5.80, *p* < .001	*t* (29) = 6.13, *p* < .001
**Road entering time (s)**	**Blocks 1-3**	*t* (24) = -7.46, *p* < .001	*t* (29) = -6.29, *p* < .001	*t* (27) = -7.26, *p* < .001	*t* (29) = -6.23, *p* < .001

		On			
		Group+1		Group-1	
		**With exclusions (N = 53)**	**Without exclusions (N = 60)**	**With exclusions (N = 53)**	**Without exclusions (N = 60)**
**Risk (1 to 10)**	**Block 3 vs. 4**	*t* (24) = -1.26, *p* = .220	*t* (29) = -0.63, *p* = .534	*t* (27) = -3.15, *p* = .004	*t* (29) = -3.18, *p* = .004
**Comprehension (1 to 10)**	**Block 3 vs. 4**	*t* (24) = 0.52, *p* = .609	*t* (29) = 0.82, *p* = .421	*t* (27) = 3.22, *p* = .003	*t* (29) = 2.95, *p* = .006
**Trust (1 to 10)**	**Block 3 vs. 4**	*t* (24) = 2.00, *p* = .057	*t* (29) = 2.21, *p* = .035	*t* (27) = 3.85, *p* < .001	*t* (29) = 3.99, *p* < .001
**Road entering time (s)**	**Block 3 vs. 4**	*t* (25) = -3.20, *p* = .004	*t* (29) = -3.03, *p* = .005	*t* (27) = -3.84, *p* < .001	*t* (29) = -4.19, *p* < .001

		On	
		Group+1 vs. Group-1	
		**With exclusions (N = 53)**	**Without exclusions (N = 60)**
**Risk (1 to 10)**	**Blocks 1-3**	*t* (51) = 1.80, *p* = .078	*t* (58) = 1.98, *p* = .053
**Comprehension (1 to 10)**	**Blocks 1-3**	*t* (51) = -2.47, *p* = .017	*t* (58) = -2.42, *p* = .019
**Trust (1 to 10)**	**Blocks 1-3**	*t* (51) = -1.69, *p* = .098	*t* (58) = -1.75, *p* = .086
**Road entering time (s)**	**Blocks 1-3**	*t* (51) = 3.07, *p* = .003	*t* (58) = 1.54, *p* = .130
**Risk (1 to 10)**	**Failure trial 1**	*t* (51) = 0.75, *p* = .456	*t* (58) = 0.72, *p* = .474
**Comprehension (1 to 10)**	**Failure trial 1**	*t* (51) = 1.50, *p* = .139	*t* (58) = 0.85, *p* = .396
**Trust (1 to 10)**	**Failure trial 1**	*t* (51) = 1.13, *p* = .265	*t* (58) = 0.96, *p* = .340
**On road when vehicle passes (no of participants)**	**Failure trial 1**	*p* = 1.000	*p* = 1.000
**Risk (1 to 10)**	**Failure trial 2**	*t* (51) = 1.47, *p* = .147	*t* (58) = 1.5, *p* = .138
**Comprehension (1 to 10)**	**Failure trial 2**	*t* (51) = 1.68, *p* = .099	*t* (58) = 1.05, *p* = .297
**Trust (1 to 10)**	**Failure trial 2**	*t* (51) = 0.63, *p* = .530	*t* (58) = 0.31, *p* = .758
**On road when vehicle passes (no of participants)**	**Failure trial 2**	*p* = .019	*p* = .005

With exclusions (N = 53)
+1 group
25 participants (12 males and 13 females) aged between 19 and 34 years (M = 24.9; SD = 3.8) took part in the study.
13 participants were used to left-hand traffic, 5 participants were used to right-hand traffic, and the remaining 7 participants were used to both left- and right-hand traffic.

-1 group
28 participants (13 males and 15 females) aged between 19 and 35 years (M = 24; SD = 4.2) took part in the study.
15 participants were used to left-hand traffic, 5 participants were used to right-hand traffic, and the remaining 8 participants were used to both left- and right-hand traffic.

Without exclusions (N = 60)
+1 group
30 participants (15 males and 15 females) aged between 18 and 34 years (M = 24.5; SD = 3.8) took part in the study.
14 participants were used to left-hand traffic, 8 participants were used to right-hand traffic, and the remaining 8 participants were used to both left- and right-hand traffic.

-1 group
30 participants (15 males and 15 females) aged between 19 and 35 years (M = 24.3; SD = 4.2) took part in the study.
15 participants were used to left-hand traffic, 7 participants were used to right-hand traffic, and the remaining 8 participants were used to both left- and right-hand traffic.

Table S2

Means of the participants' results per dependent measure and experimental condition (full sample, N = 60)

		Group +1					Group -1				
		On 33 m	Off 33 m	On 43 m	Off 43 m	Off NY	On 33 m	Off 33 m	On 43 m	Off 43 m	Off NY
Risk (1 to 10)	Block 1	4.18	5.00	3.27	4.80	6.13	3.22	5.17	2.73	5.10	5.24
	Block 2	3.97	4.20	3.09	4.33	5.93	3.04	5.00	2.37	4.93	4.95
	Block 3	3.76	4.47	2.86	4.23	6.11	2.64	5.00	2.20	4.70	4.88
	Failure trial 1	9.03					8.67				
	Block 4	3.76	4.03	3.04	4.20	5.82	3.42	4.60	2.70	4.27	4.53
	Failure trial 2	8.73					7.83				
Comprehension (1 to 10)	Block 1	6.62	5.87	7.80	6.03	6.40	7.76	5.03	8.13	5.57	6.37
	Block 2	6.94	5.87	7.97	6.03	6.51	8.07	5.53	8.63	6.13	6.25
	Block 3	7.06	6.13	8.23	6.50	6.38	8.22	5.57	8.63	6.10	6.60
	Failure trial 1	2.93					2.33				
	Block 4	7.09	6.50	7.92	6.47	6.73	7.44	6.07	8.11	6.20	6.65
	Failure trial 2	3.57					2.87				
Trust (1 to 10)	Block 1	6.40	5.80	7.52	5.93	5.49	7.38	5.40	7.87	5.73	5.72
	Block 2	6.79	5.90	7.80	6.03	5.62	7.63	5.60	8.27	6.07	5.85
	Block 3	6.76	6.07	7.94	6.27	5.48	7.87	5.47	8.40	5.57	5.99
	Failure trial 1	2.20					1.73				
	Block 4	6.52	6.17	7.21	6.30	5.49	6.49	5.37	7.41	5.97	5.94
	Failure trial 2	1.97					1.83				
Road entering (s)	Block 1	6.58	7.28	6.24	7.21		6.05	7.44	5.68	7.45	
	Block 2	6.57	6.94	6.21	7.23		5.69	7.38	5.26	7.23	
	Block 3	6.46	6.97	6.06	7.10		5.69	7.24	5.16	7.27	
	Block 4	6.77	7.13	6.58	7.18		6.70	7.63	5.71	7.62	

Note. The cells for Risk, Comprehension, and Trust are linearly filled according to the depicted mean value on a scale from 1 (no orange in the cell) to 10 (entire cell orange). The cells for the road entering time are linearly filled according to the depicted mean value on a scale from 5 s to 8 s. NY = no yielding.

Table S3

Standard deviations of the participants' results per dependent measure and experimental condition (after exclusion of participants, N = 53)

		On 33 m	Off 33 m	On 43 m	Off 43 m	Off NY	On 33 m	Off 33 m	On 43 m	Off 43 m	Off NY
**Risk (1 to 10)**	**Block 1**	1.76	2.16	1.55	2.45	2.93	2.09	2.43	2.05	2.48	2.97
	**Block 2**	1.73	2.15	1.90	2.11	3.26	1.90	2.34	1.87	2.51	2.98
	**Block 3**	1.95	2.25	1.81	2.56	3.00	1.83	2.50	1.64	2.60	3.05
	**Failure trial 1**	1.73					2.11				
	**Block 4**	2.10	1.78	1.84	2.30	3.05	2.07	2.45	1.95	2.35	2.61
	**Failure trial 2**	1.71					2.88				
**Comprehension (1 to 10)**	**Block 1**	1.61	1.60	1.90	2.17	2.44	1.59	2.28	1.39	2.28	2.42
	**Block 2**	1.57	2.38	1.86	2.24	2.47	1.56	2.62	1.21	2.32	2.74
	**Block 3**	1.57	2.15	1.27	2.50	2.20	1.63	2.53	1.38	2.47	2.69
	**Failure trial 1**	3.13					2.04				
	**Block 4**	1.38	2.12	1.47	2.12	2.21	1.78	2.03	1.52	2.05	2.60
	**Failure trial 2**	3.01					1.89				
**Trust (1 to 10)**	**Block 1**	1.89	1.88	2.09	1.88	2.43	1.84	2.17	1.58	2.33	2.63
	**Block 2**	1.82	1.79	1.95	1.96	2.68	1.78	2.57	1.53	2.41	2.84
	**Block 3**	1.86	1.76	1.70	2.25	2.59	1.70	2.63	1.47	2.39	2.77
	**Failure trial 1**	2.38					1.03				
	**Block 4**	1.75	1.68	2.00	1.89	2.63	2.13	2.09	1.84	2.27	2.59
	**Failure trial 2**	1.96					1.30				
**Road entering (s)**	**Block 1**	1.15	0.72	1.65	1.45		1.61	1.87	2.06	1.99	
	**Block 2**	1.18	1.17	1.71	1.57		1.84	1.44	2.19	2.20	
	**Block 3**	1.33	1.15	1.79	1.67		1.71	1.23	2.01	1.49	
	**Block 4**	1.24	1.14	1.74	1.68		1.43	1.05	2.02	1.63	

Note. The cells are linearly filled according to the depicted standard deviation on a scale from 0 (no orange in the cell) to 3 (entire cell orange). NY = no yielding.

The presence questionnaire consisted of 20 questions. The table below shows the means and standard deviations of the responses.

Table S4

Mean and standard deviations (SD) of responses to the Presence Questionnaire (full sample, N = 60)

	**Question**	**Mean**	***SD***
**1**	How proficient in moving and interacting with the virtual environment did you feel at the end of the experience? (1: Not proficient; 7: Very proficient)	6.40	0.72
**2**	How well could you concentrate on the assigned tasks or required activities rather than on the mechanisms used to perform those tasks or activities? (1: Not at all; 7: Completely)	6.17	1.11
**3**	How closely were you able to examine objects? (1: Not at all; 7: Very closely)	5.57	1.33
**4**	How much did the auditory aspects of the environment involve you? (1: Not at all; 7: Completely')	3.18	2.20
**5**	How quickly did you adjust to the virtual environment experience? (1: Not at all; 7: Less than one minute)	6.08	1.03
**6**	How natural was the mechanism which controlled movement through the environment? (1: Extremely artificial; 7: Completely natural)	5.82	1.10
**7**	Were you able to anticipate what would happen next in response to the actions that you performed? (1: Not at all; 7: Completely)	5.42	1.38
**8**	How responsive was the environment to actions that you initiated (or performed)? (1: Not responsive; 7: Completely responsive)	5.70	1.18
**9**	How involved were you in the virtual environment experience? (1: Not involved; 7: Completely engrossed)	6.03	0.90
**10**	How natural did your interactions with the environment seem? (1: Extremely artificial; 7: Completely natural)	5.72	1.34
**11**	How compelling was your sense of objects moving through space? (1: Not at all; 7: Very compelling)	6.13	0.93
**12**	How much did the visual aspects of the environment involve you? (1: Not at all; 7: Completely)	5.65	1.18
**13**	How much delay did you experience between your actions and expected outcomes? (1: No delays; 7: Long delays)	2.43	1.60
**14**	How much did your experiences in the virtual environment seem consistent with your real world experiences? (1: Not consistent; 7: Very consistent)	5.62	1.22
**15**	How completely were you able to actively survey or search the environment using vision? (1: Not at all; 7: Completely)	6.00	0.97
**16**	How much did the control devices/ motion sensors interfere with the performance of assigned tasks or with other activities? (1: Not at all; 7: Interfered greatly)	2.52	1.70
**17**	How well could you examine objects from multiple viewpoints? (1: Not at all; 7: Extensively)	5.42	1.27
**18**	How compelling was your sense of moving around inside the virtual environment? (1: Not compelling; 7: Very compelling)	6.05	1.02
**19**	How much did the visual display quality interfere or distract you from performing assigned tasks or required activities? (1: Not at all; 7: Required task performance)	2.13	1.23
**20**	How much were you able to control events? (1: Not at all; 7: Completely)	4.42	1.67

Table S5

Standard deviations of the participants' results per dependent measure and experimental condition (full sample, N = 60)

		Group +1					Group -1				
		On 33 m	Off 33 m	On 43 m	Off 43 m	Off NY	On 33 m	Off 33 m	On 43 m	Off 43 m	Off NY
**Risk (1 to 10)**	**Block 1**	1.76	2.27	1.75	2.37	2.85	2.04	2.35	2.00	2.41	2.94
	**Block 2**	1.78	1.99	1.80	2.01	3.15	1.84	2.27	1.81	2.49	3.00
	**Block 3**	1.87	2.11	1.71	2.42	2.89	1.76	2.45	1.59	2.64	3.00
	**Failure trial 1**	1.87					2.07				
	**Block 4**	2.02	1.75	1.73	2.17	3.02	2.02	2.37	1.89	2.38	2.62
	**Failure trial 2**	1.72					2.79				
**Comprehension (1 to 10)**	**Block 1**	1.58	1.87	1.85	2.19	2.47	1.58	2.24	1.37	2.22	2.34
	**Block 2**	1.55	2.26	1.76	2.28	2.52	1.51	2.56	1.20	2.26	2.65
	**Block 3**	1.59	2.05	1.20	2.35	2.36	1.60	2.49	1.35	2.38	2.60
	**Failure trial 1**	3.04					2.35				
	**Block 4**	1.39	2.00	1.58	2.21	2.32	1.73	2.03	1.48	2.02	2.53
	**Failure trial 2**	2.93					2.16				
**Trust (1 to 10)**	**Block 1**	2.00	2.06	2.19	2.02	2.42	1.79	2.13	1.53	2.27	2.57
	**Block 2**	1.97	1.86	2.03	1.97	2.71	1.73	2.54	1.48	2.33	2.75
	**Block 3**	1.93	1.80	1.70	2.24	2.67	1.66	2.60	1.42	2.34	2.70
	**Failure trial 1**	2.38					1.17				
	**Block 4**	2.00	1.97	2.25	2.07	2.84	2.13	2.04	1.80	2.27	2.56
	**Failure trial 2**	1.90					1.39				
**Road entering (s)**	**Block 1**	2.09	2.09	2.13	2.43		1.65	2.15	2.09	2.25	
	**Block 2**	2.02	2.32	2.17	2.53		1.83	1.78	2.12	2.37	
	**Block 3**	2.06	2.28	2.29	2.38		1.75	1.63	1.95	1.83	
	**Block 4**	2.10	2.04	2.20	2.45		1.41	1.02	1.97	1.65	

Note. The cells are linearly filled according to the depicted standard deviation on a scale from 0 (no orange in the cell) to 3 (entire cell orange). NY = no yielding.

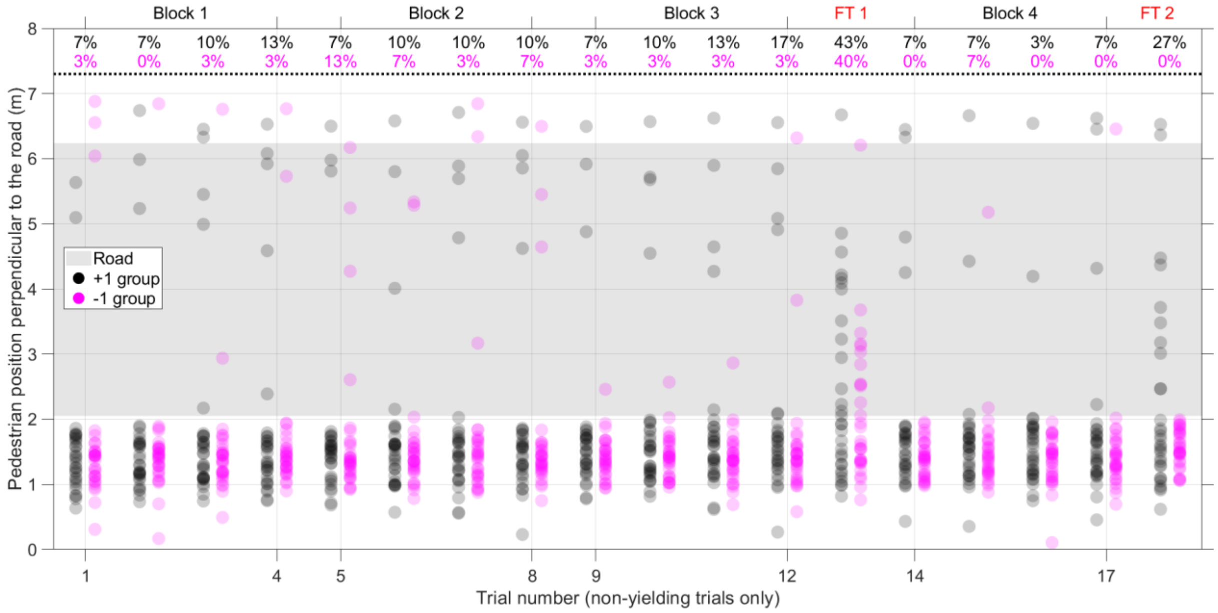

Figure S1. Pedestrian location at the moment the front of the car passed for the full sample ($N = 60$). The top of the figure shows the percentage of participants in the +1 Group (30 participants) and -1 Group (30 participants) who were on the road at the moment the car passed. The markers are transparent so that overlap can be distinguished. FT 1 = Failure Trial 1, FT 2 = Failure Trial 2.
